# Three‐dimensional quantitative dose reduction analysis in MammoSite balloon by Monte Carlo calculations

**DOI:** 10.1120/jacmp.v8i4.2669

**Published:** 2007-11-20

**Authors:** Zhengdong Zhang, E. Ishmael Parsai, John J. Feldmeier

**Affiliations:** ^1^ University of Toledo, College of Medicine Department of Radiation Oncology Toledo Ohio U.S.A.

**Keywords:** MammoSite, HDR, Monte Carlo, brachytherapy, TG‐43U1

## Abstract

Current treatment planning systems (TPSs) for partial breast irradiation using the MammoSite brachytherapy applicator (Cytyc Corporation, Marlborough, MA) often neglect the effect of inhomogeneity, leading to potential inaccuracies in dose distributions. Previous publications either have studied only a planar dose perturbation along the bisector of the source or have paid little attention to the anisotropy effect of the system. In the present study, we investigated the attenuation‐corrected radial dose and anisotropy functions in a form parallel to the updated American Association of Physicists in Medicine TG‐43 formalism. This work quantitatively delineates the inaccuracies in dose distributions in three‐dimensional space. Monte Carlo N‐particle transport code simulations in coupled photon–electron transport were used to quantify the changes in dose deposition and distribution caused by the increased attenuation coefficient of iodine‐based contrast solution. The source geometry was that of the VariSource wire model VS2000 (Varian Medical Systems, Palo Alto, CA). The concentration of the iodine‐based solution was varied from 5% to 25% by volume, a range recommended by the balloon's manufacturer. Balloon diameters of 4, 5, and 6 cm were simulated. Dose rates at the typical prescription line (1 cm away from the balloon surface) were determined for various polar angles. The computations showed that the dose rate reduction throughout the entire region of interest ranged from 0.64% for the smallest balloon diameter and contrast concentration to 6.17% for the largest balloon diameter and contrast concentration. The corrected radial dose function has a predominant influence on dose reduction, but the corrected anisotropy functions explain only the effect at the MammoSite system poles. By applying the corrected radial dose and anisotropy functions to TPSs, the attenuation effect can be reduced to the minimum.

PACS number: 87.53.‐j

## I. INTRODUCTION

The MammoSite Radiation Therapy System (Cytec Corporation, Marlborough, MA) is a new, minimally invasive method of delivering internal radiation therapy following lumpectomy for breast cancer.^(^ ^1^ ^–^ ^9^ ^)^ Therapy is given on an outpatient basis and can be completed in 5 days.

The general physical guidelines for determining whether the MammoSite system is appropriate for a specific patient include assessment of balloon conformance to the lumpectomy cavity to determine the adequacy of target coverage and dose distribution, minimum skin distance from the balloon surface, and balloon diameter and symmetry. Accurate positioning of the high dose rate (HDR) source at the center of the balloon or a specific dwell site is an essential part of the procedure. Small perturbations of the source position can result in significant variations in dose at the prescription line. Balloon integrity is also important, and therefore verification radiographs or ultrasound imaging are used at each fraction to ensure balloon deformation or deflation. The radiopaque contrast used inside the balloon enhances image quality and contributes to treatment planning and quality assurance of the procedure.^(10)^

In the United States, ${}^{192}\text{Ir}$ is being widely used as a HDR source. It emits a wide spectrum (0.11 MeV – 1.378 MeV), including many low‐energy components.^(11)^ Because of the photoelectric effect, low‐energy photons are preferentially absorbed by high‐*Z* media. Because contrast materials typically contain elements with high atomic numbers—for example, iodine (Z=53), the balloon content cannot be considered tissue‐ or water‐equivalent.

Currently, most of the widely used treatment planning systems (TPSs) for brachytherapy are based on dosimetry in water and do not take variations in attenuation into account. Thus, patients may receive less dose than planned. Use of a small‐volume parallel‐plate ion chamber to take measurements of dose perturbations resulting from the use of contrast material in the balloon have recently been reported.^(^ ^12^ ^–^ ^14^ ^)^ The use of Monte Carlo simulation with a range of balloon diameters and radiopaque contrast concentrations to determine contrast effects on dosimetry along the source bisect has been reported for the Nucletron microSelectron HDR v2 ${}^{192}\text{Ir}$ source (Nucletron, Veenendaal, Netherlands).^(10)^

Because of the generally increased use of this new procedure, we here report a dosimetric study of the effects of contrast in the balloon over the entire azimuthal space. This work used Monte Carlo simulations for a range of balloon diameters and radiopaque contrast concentrations with the VariSource HDR ${}^{192}\text{Ir}$ source (Varian Medical Systems, Palo Alto, CA) currently in use in our radiotherapy center. To incorporate contrast attenuation of this kind into the TPS, we suggest using attenuation‐corrected radial dose functions and anisotropy functions in a form that parallels the American Association of Physicists in Medicine (AAPM) TG‐43U1 formalism.^(15)^

## II. MATERIALS AND METHODS

### A. Description of source and balloons

The phantom used in this Monte Carlo simulation was a sphere, 30 cm in diameter, containing water (composition: 11.2% hydrogen and 88.8% oxygen by weight). The balloon was assumed to be a sphere positioned at the center of the water phantom. The silicone balloon wall and nylon catheter of the source were not modeled.

We simulated three balloon diameters (4, 5, and 6 cm) to cover a preponderance of the clinical applications per the manufacturer's recommendation and indications as approved by the U.S. Food and Drug Administration. The composition of the material inside the balloon was assumed to be an iodine‐based radiopaque material mixed with saline. The composition of the radiopaque material was modeled based on Amersham Omnipaque (molecular chemical structure: 821.14 molecular weight, 46.36% iodine content by weight, and density 1.406 g ${\text{cm}}^{-3}$; Amersham Health, Little Chalfont, U.K.). The contrast concentrations modeled in the study were 5%, 10%, 15%, 20%, and 25% by volume, which cover the range recommended by the manufacturer. The contrast solution densities and the elemental composition by weight of the simulated contrast solutions are listed in detail in Kassas et al.^(10)^

**Figure 1:**
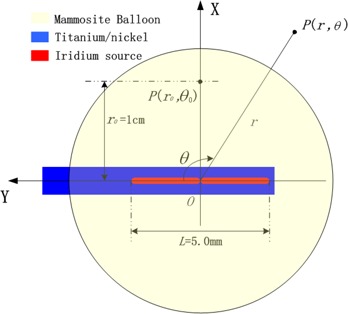
Schematic of the MammoSite assembly and the coordinate system used in the simulation.

The source simulated in this study is the VariSource HDR ${}^{192}\text{Ir}$ source (model VS2000: Varian Medical Systems, Palo Alto, CA), which has been described in detail by Angelopoulos et al.^(16)^ The energy spectrum of this ${}^{192}\text{Ir}$ source is taken from Glasgow and Dillman.^(11)^ Fig. 1 shows a schematic of the balloon with the source and the coordinate system used in the simulation.

### B. Monte Carlo calculations

The Monte Carlo N‐particle transport code (MCNP), version 4ca1, used in this study is a general‐purpose code capable of simulating neutron, photon, and electron transport in a three‐dimensional (3D), heterogeneous geometry system.^(17)^ The simulation of photon transport includes photoelectric absorption with the possibility of fluorescent emission, coherent and incoherent scattering, and pair production. The continuous slowing‐down model used for electron transport includes positrons, *k* X‐rays, and bremsstrahlung.

In the present study, the primary photons are produced isotropically and uniformly through the source core. A photon history is terminated if the photon's energy falls below a selected cutoff value (in this work, 10 keV). The ${}^{192}\text{Ir}$ source was positioned at the center of a spherical liquid water phantom 30 cm in diameter to allow for comparison with similar studies.^(16)^ The coordinate system used in the simulation was the same as that introduced by Angelopoulos,^(16)^ in which the origin *O* coincides with the center of the radioactive core (between the two seeds), and the positive *y* axis aligns with the wire side of the source drive. The polar angle θ=0 degrees is defined as the proximal (drive wire) side of the source, and the polar angle θ=180 degrees corresponds with the distal end of the source. The Ti–Ni wire was not simulated in its entire usable length, but only within the 30‐cm spherical phantom. The phantom sphere was divided into discrete concentric spherical shells (grid) of 1 mm, each split into angular intervals of 1 degree both with respect to polar angle θ (0, Π) and azimuthal angle φ (0, 2 Π). However, because all dosimetric quantities involved in the present study are isotropic with respect to azimuthal angle φ, this 3D segmentation can be simplified to a two‐dimensional (2D) segmentation only with respect to coordinates *r* (ranging from 0.2 mm to 150 mm, in 1‐mm intervals) and θ (ranging from 0 degrees to 180 degrees in 1‐degree intervals).

The MCNP code was used to calculate the dose rate of the source at any point of interest in the phantom. The range of the grid (tally mesh) chosen was sufficient to cover a distance well beyond the typical prescription line, and it allowed adequate backscatter within the phantom. Compton scattering, photoelectric effect, and coherent scattering were accounted for in the MCNP4ca1 simulation. Characteristic X‐rays following photoelectric absorption were also included in the calculations. Because of the low energies of photons emitted by the ${}^{192}\text{Ir}$ source, secondary charged particle equilibrium can be assumed to exist, and therefore absorbed dose can be approximated by collision kerma.^(18)^ To keep the uncertainty in the Monte Carlo calculations low, $3\times 10^{8}$ histories were used for each MCNP simulation. A separate simulation, in which the source was centered in a sphere 5 m in diameter with a composition of dry air, was used with the MCNP to calculate the air kerma strength. The air kerma strength was calculated at 1 m from the center of the source. The grid used for the calculation had a larger scoring bin step of 1 cm so as to achieve adequate uncertainty in the Monte Carlo calculations.

### C. Attenuation corrections in the presence of MammoSite balloons

The AAPM TG‐43U1 formalism describes 2D dose distributions in water‐equivalent homogeneous media for a cylindrically symmetric radioactive source. Provided that cylindrical symmetry is maintained, the general form of the TG‐43U1 formalism can be extended to include a source–balloon system. Strict adherence to the AAPM TG‐43U1 protocol for a MammoSite system would require calibration of the source–balloon system by the approved calibration laboratories to obtain the air kerma strength of the assembly. The dose rate constant and, finally, the radial and anisotropy functions of the system would then have to be computed. Because such a procedure would be costly and impractical, we chose to maintain the original values of air kerma strength $\left(S_{k}\right)$ and dose rate constant (Λ), and to modify only the radial function $g_{\text{corr}}(r)$ and the anisotropy function $F_{\text{corr}}(r,\theta )$ to take the contrast attenuation into account. The dose rate in the presence of the balloon, $D_{\text{corr}}(r,\theta )$, then becomes

(1) $${\dot{D}}_{corr}(r,\theta )=S_{K}\Lambda \frac{G(r,\theta )}{G(1\text{cm},\pi /2)}g_{corr}(r)F_{corr}(r,\theta ),$$

with

(2) $$\Lambda =\frac{\overset{\cdot }{D}(1\text{cm},\pi /2)}{S_{K}},$$

(3) $$G(r,\theta )=\frac{\beta }{Lr\sin\theta },$$

and

(4) $$g_{corr}(r)=\frac{{\dot{D}}_{corr}(r,\pi /2)G(1\text{cm},\pi /2)}{\dot{D}(1\text{cm},\pi /2)G(r,\pi /2)},$$

where $\dot{D}(1\;\text{cm},\Pi /2)$ is the dose rate in water.

(5) $$F_{corr}(r,\theta )=\frac{{\dot{D}}_{corr}(r,\theta )G(r,\pi /2)}{{\dot{D}}_{corr}(r,\pi /2)G(r,\theta )}.$$

### D. Experimental verification of applicator attenuation

**Figure 2:**
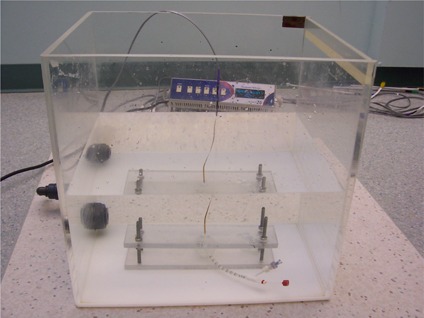
Experimental setup for measurement by MOSFET (metal oxide semiconductor field effect transistor).

We made MOSFET (metal oxide semiconductor field effect transistor) point measurements in a water tank of dimension $32\times 39\times 40\;{\text{cm}}^{3}$. Readings were taken 1 cm away from the surface of balloon along the transverse plane of the source. A special homemade plastic holder (Fig. 2) kept the balloon assembly and two identical MOSFET dosimeters (model TN‐502RD: Thomson and Nielsen Electronics, Ottawa, Canada) in well‐defined positions.

In all measurements, the source was connected to the HDR unit and delivered to the center of the balloon to mimic the exact simulation conditions. The MOSFETs were taped onto a water‐equivalent slab of 1 cm thickness (mimicking a typical prescription point located 1 cm from the balloon surface) with their axes parallel to the source axis. By taking the average of the two MOSFET readings, the effect of potential errors attributable to the uncertainties in the source position was significantly reduced.

Dose attenuation was measured by taking the ratio of the averaged MOSFET readings with and without the desired contrast filling the balloon. Great care was taken not to perturb the positions of the MOSFETs or the balloon assembly when solutions were changed. All measurements were taken within a few hours to minimize change in the ambient conditions and reduction in source activity.

## III. RESULTS AND DISCUSSION

### A. Contrast effects

**Figure 3:**
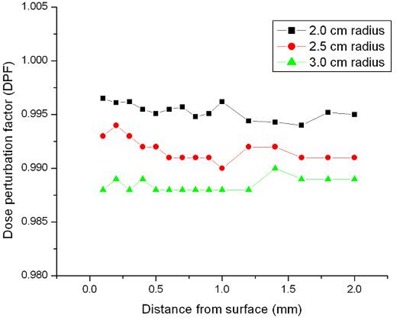
Monte Carlo simulation showing dose perturbation as a function of the distance from the surface of the balloon for three different balloon radii and 5% concentration.

Table 1 provides the results of the average dose rate reduction at 1 cm away from the balloon surface, the typical prescription line, for balloon diameters 4, 5, and 6 cm and contrast concentrations 5%, 10%, 15%, 20%, and 25% by volume. The dose rate reduction throughout the entire region of interest between polar angles 0 degrees and 180 degrees ranged from 0.64% for the smallest balloon diameter and contrast concentration, to 6.17% for the largest balloon diameter and contrast concentration. These results agree in general with the data from Kassas et al.,^(10)^ who reported a simulated dose rate reduction along the source bisector with the Nucletron microSelectron HDR ${}^{192}\text{Ir}$ source. The larger the balloon, therefore, the higher the perturbation in the dose rate at the prescription line when high‐concentration contrast was used. Meanwhile, the dose enhancement reported by Cheng et al.^(13)^ was not observed in our study at the surface or in the immediate vicinity of the MammoSite for balloon diameters of 4, 5, and 6 cm and for contrast concentrations of 5% and 15%. Fig. 3 shows the simulation results for the 5% concentration.

**Table 1:** Percentage reduction (Δ%) in average dose rate at 1 cm from the balloon because of contrast, relative to water, for the various balloon diameters

		Percentage reduction (Δ%) at contrast concentration			
Balloon diameter (cm)	5%	10%	15%	20%	25%
4	−0.64	−1.41	−2.16	−2.90	−3.51
5	−1.00	−2.07	−3.06	−4.02	−4.80
6	−1.41	−2.80	−4.04	−5.23	−6.17

### B. Azimuth effects

**Figure 4:**
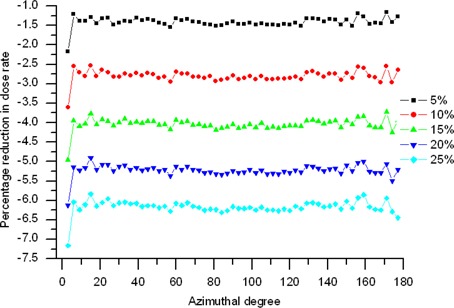
Monte Carlo simulation showing variation of the dose rate reduction as a function of iodine concentration for a balloon 6 cm in diameter.

Fig. 4 shows the variations in dose rate reduction as a function of polar angles for a balloon diameter of 6 cm. For the same balloon diameter and contrast concentration, the variations fluctuate very little for most of the azimuth range, except on the proximal (drive wire) side of the source. In addition, the variations for the other balloon sizes and contrast concentrations show the same trend over polar angles. From the figure, the area near 0 degrees can be seen to experience a larger dose reduction against the average variations. The dose degradation along the source axis occurs because of the effect of oblique filtration and self‐absorption.^(14)^

### C. Spectral variations

**Figure 5:**
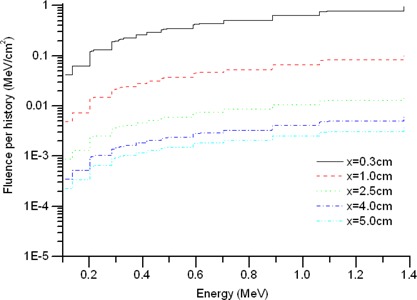
Monte Carlo–generated photon spectra for the source in water at various distances along the transverse plane.

The scattering of primary photons and emission of fluorescent X‐rays by the source matrix, the Ti–Ni alloy encapsulation, and the contrast solution broadens the originally discrete photon spectrum, which therefore spans the entire energy range below the maximum photon energy of ${}^{192}\text{Ir}$. Fig. 5 shows the Monte Carlo–generated photon spectra for the bare source at various distances. The energy‐dependent fluences are normalized per primary photon history. Because of the high absorption coefficients at such low energies, photon fluences below 0.3 MeV decrease rapidly with increasing distance from the source, and the photon spectra between 0.3 MeV and 1.4 MeV decrease slowly and uniformly because of the relatively low and constant absorption coefficients in that energy range.

**Figure 6:**
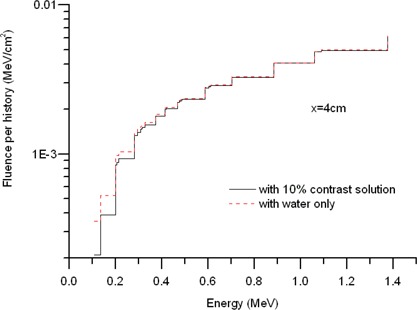
Monte Carlo–generated photon spectra, showing spectral variations resulting from the presence of the contrast at the reference distance of 4 cm.

The introduction of high‐*Z* contrast material affects the photon spectra. Fig. 6 shows these spectral variations for a 10% contrast concentration, in a balloon of radius 3 cm, at the reference distance of 4 cm away from the center. Low‐energy photons of E<0.4 MeV are absorbed preferentially by the iodine contained in the balloon. Thus, the photon fluences of the balloon in this energy range are lowered—that is, spectrum hardening occurs.

### D. Air kerma strength per unit activity, Sk/A, and dose rate constant, Λ

In Table 2, the calculated values of air kerma strengths per unit source activity are shown at several transverse distances (2 – 100 cm) from the center of the source, and a comparison with previous studies is made. The data of Angelopoulos et al.^(16)^ were calculated by the same MCNP simulations using the primary photon spectra of Glasgow and Dillman^(11)^ (same as ours). They were the average values at distances ranging from 2 cm to 100 cm in 1‐cm intervals in dry air and at distances ranging from 2 cm to 30 cm in 1‐cm intervals in free space.

**Table 2:** Comparison with published data of calculated air kerma strengths per unit activity in dry air

Transverse distance *x*(cm)	Present study $S_{k}/A(10^{-8}U/\text{Bq})$	Angelopoulos et al.^(^ ^16^ ^,^ ^a^ ^)^ $S_{k}/A(10^{-8}U/\text{Bq})$
5	10.22±0.05%	
10	10.25±0.05%	10.27±0.05(in free space)
20	10.26±0.05%	
50	10.26±0.05%	10.28±0.05(in air)
80	10.26±0.05%	
100	10.26±0.05%	

a The cutoff energy of photon fluence spectra was 10 keV.

Our results showed excellent agreement with the published data. The air kerma strength per unit activity was determined $(10.26\pm 0.05\%)10^{-8}U/\text{Bq}(\mu \text{Gy}\;m^{2}h^{-1}{\text{Bq}}^{-1}$) and was found to be almost constant in the region 10 – 100 cm. To allow for comparisons with published data, we also calculated the dose rate constant Λ of the ${}^{192}\text{Ir}$ source, which is used to convert the in‐air source strength to dose in water. We computed the average value of air kerma at points on the transverse plane 2 – 100 cm from the source center, and the dose in water at 1‐cm transverse distance. The dose rate constant was determined to be $1.097\pm 0.05\;\text{cGy}h^{-1}U^{-1}$. That result agreed with published data^(16)^ within statistical uncertainties. Note that the constant does not include any effects of the contrast.

### E. Attenuation‐corrected radial dose functions, $g_{\text{corr}}(r)$

**Figure 7:**
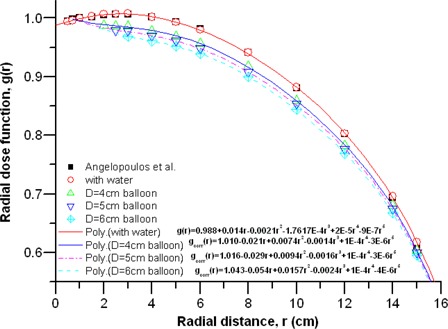
Radial dose functions calculated for source in water only and source in 15% concentration of balloon with various diameters and comparisons to published data. Fifth‐order polynomial fittings to our calculated radial dose functions are also presented.

Using equation 4, we calculated the attenuation‐corrected radial dose functions for the three diameters of balloons containing a 15% contrast concentration. Because another material, contrast, was introduced into the medium along the transverse axis, the difference in the functions appeared significantly as shown. Table 3 and Fig. 7 give the results of the present work and the previously published data^(16)^ for water only. The radial dose functions were fitted to a fifth‐order polynomial:

**Table 3:** Comparison of radial dose functions, g(r) or $g_{\text{corr}}$ (r), from various Monte Carlo calculations

	Present work				Angelopoulos et al.^(16)^
*r* (cm)	Water only	D4‐cm balloon	D5‐cm balloon	D6‐cm balloon	Water only
0.5	0.994	N/A	N/A	N/A	0.995
0.7	0.996	N/A	N/A	N/A	0.998
1.0	1.000	N/A	N/A	N/A	1.000
1.5	1.003	N/A	N/A	N/A	1.002
2.0	1.007	0.988	N/A	N/A	1.005
2.5	1.007	0.986	0.979	N/A	1.006
3.0	1.008	0.986	0.978	0.969	1.006
4.0	1.002	0.978	0.970	0.961	1.002
5.0	0.993	0.969	0.961	0.952	0.993
6.0	0.980	0.957	0.948	0.939	0.981
8.0	0.941	0.917	0.908	0.900	0.941
10.0	0.882	0.860	0.853	0.844	0.881
12.0	0.803	0.783	0.776	0.769	0.803
14.0	0.696	0.680	0.675	0.668	0.693
15.0	0.618	0.604	0.600	0.595	0.609

(6) $$g(r)=a_{5}r^{5}+a_{4}r^{4}+a_{3}r^{3}+a_{2}r^{2}+a_{1}r^{1}+a_{0}.$$

Fig. 7 shows the fit parameters. Deviations of individual dose points from the polynomial are within the statistical uncertainties (1σ) of the Monte Carlo calculations, which are less than ±0.1% at r ≤; 15 cm.

### F. Attenuation‐corrected anisotropy functions, Fcorr (r, θ)

**Figure 8:**
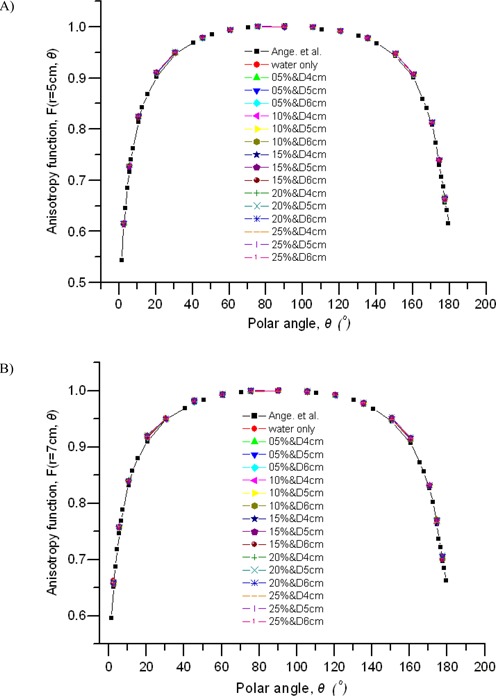
Anisotropy functions at two radial distances calculated for source in water and for source in various balloons, and their comparisons to published data. Angle 0 degrees is toward a source delivery cable, and angle 180 degrees is toward the distal end of a source.

The attenuation‐corrected anisotropy functions for the system were calculated using equation 5. The graphs in Fig. 8 show comparisons measured 5 cm and 7 cm away from the source center for balloons of various diameters and contrast concentrations. Comparisons with published data^(16)^ for the source in water only are also shown. Our results for the source in water agree with the data of Angelopoulos et al.^(16)^ within 0.9% for polar angles between 6 degrees and 177 degrees. For angles at or close to the MammoSite poles (0 degrees and 180 degrees), the model will not accurately represent the balloon system, because the shape of the sphere at these angles is a variable function of balloon size and pressure at its surface. However, the results presented here agree favorably in all other angles subtended by the model. The anisotropy function $F_{F}\;(r,\theta )$ accounts for the angular dependence of photon absorption and scatter in the encapsulation and the medium, and the addition of evenly dispersed contrast solution to the balloon causes no change at all in the state of angular distribution of photon absorption and scattering. As a result, the anisotropy functions fluctuate very little in the presence of the balloons, as Fig. 8 clearly shows. At r=7 cm and θ> 4 degrees, the differences in the attenuation‐corrected anisotropy functions between the source plus water and the source plus various balloons are less than 0.1%. However, near the proximal end of the source, where the large filtration and self‐absorption are accessible, the differences become significant, reaching about 1%. That finding also explains the sharp drop of dose rate reduction at θ< 4 degrees in Fig. 4.

We also calculated the anisotropy functions at r=5 cm. As *r* decreases, the anisotropy characteristics become smaller.

### G. Experimental verification of dose reductions in the presence of contrast

The dose reduction attributable to attenuation from the contrast solution was confirmed by MOSFET measurements. Table 4 shows the comparison between the Monte Carlo calculations and the measurements along the transverse plane in the presence of contrast at various concentrations. The values in each column are expressed as percentages of the doses for source with water only, under the same conditions. The dose reductions calculated by the Monte Carlo method agreed to within 0.7% with the reductions measured by MOSFET. The measurement uncertainty in this experiment was within ±3%.

**Table 4:** Monte Carlo–computed dose reduction and dose reduction measured by MOSFET (metal oxide semiconductor field effect transistor) by addition of two contrasts, expressed as a percentage of the dose of source with water only

	D4‐cm balloon		D5‐cm balloon		D6‐cm balloon	
Contrast concentration	Monte Carlo	Measured	Monte Carlo	Measured	Monte Carlo	Measured
10%	98.6	99.1	97.9	98.6	97.2	97.8
15%	97.8	98.2	96.9	97.5	96.0	96.7

## IV. CONCLUSIONS

Because the mass attenuation coefficient is large for low energies and high atomic number media (because of the predominance of photoelectric interactions under these conditions), the dose reduction attributable to contrast attenuation cannot simply be ignored. It can be expressed in the decrease of radial dose functions along the transverse plane and the decrease of anisotropy functions at oblique angles from the transverse plane. Through quantitative analysis using Monte Carlo methods, we find that the radial dose functions have a predominant influence on dose reduction, but that the anisotropy functions play only a small role in explaining the obvious difference at the proximal end of the source.

The potential for a 5% improvement in accuracy would be worth the effort. Considering that current treatment planning systems are using the AAPM TG‐43U1 formalism, replacing the radial and anisotropy functions of source in water with the corresponding parameters of the source–balloon assembly should not require major changes to the computational algorithm.
